# Visceral Adiposity, Anthropometric and Liver Function Indexes for Identifying Metabolic Dysfunction Associated Steatotic Liver Disease (MASLD) in Adolescents with Obesity: Which Performs Better?

**DOI:** 10.3390/jcm14062085

**Published:** 2025-03-19

**Authors:** Lara Mari, Stefano Lazzer, Alessandro Gatti, Mattia D’Alleva, Simone Zaccaron, Jacopo Stafuzza, Enrico Rejc, Matteo Vandoni, Adele Bondesan, Diana Caroli, Francesca Frigerio, Laura Abbruzzese, Enrica Ventura, Alessandro Sartorio

**Affiliations:** 1 Department of Medicine, University of Udine, 33100 Udine, Italy; mari.lara@spes.uniud.it (L.M.); mattia.dalleva@uniud.it (M.D.); simone.zaccaron@uniud.it (S.Z.); jacopo.stafuzza@uniud.it (J.S.); enrico.rejc@uniud.it (E.R.); 2 School of Sport Sciences, University of Udine, 33100 Udine, Italy; 3 Laboratory of Adapted Motor Activity (LAMA), Department of Public Health, Experimental Medicine and Forensic Science, University of Pavia, 27100 Pavia, Italy; alessandro.gatti08@universitadipavia.it (A.G.); matteo.vandoni@unipv.it (M.V.); 4 National PhD Programme in One Health Approaches to Infectious Diseases and Life Science Research, Department of Public Health, Experimental and Forensic Medicine, University of Pavia, 27100 Pavia, Italy; 5 Department of Neurosciences, Biomedicine and Movement Sciences, University of Verona, 37129 Verona, Italy; 6 Experimental Laboratory for Auxo-Endocrinological Research, Istituto Auxologico Italiano, Istituto di Ricovero e Cura a Carattere Scientifico (IRCCS), 28824 Piancavallo-Verbania, Italy; a.bondesan@auxologico.it (A.B.); d.caroli@auxologico.it (D.C.); f.frigerio@auxologico.it (F.F.); sartorio@auxologico.it (A.S.); 7 Division of Auxology, Istituto Auxologico Italiano, Istituto di Ricovero e Cura a Carattere Scientifico (IRCCS), 28824 Piancavallo-Verbania, Italy; l.abbruzzese@auxologico.it; 8 Division of Eating and Nutrition Disorders, Istituto Auxologico Italiano, Istituto di Ricovero e Cura a Carattere Scientifico (IRCCS), 28824 Piancavallo-Verbania, Italy; e.ventura@auxologico.it

**Keywords:** obesity, MASLD, fatty liver disease, adolescents

## Abstract

**Background:** Metabolic Dysfunction Associated Steatotic Liver Disease (MASLD) is the accumulation of fat in the liver without excessive alcohol consumption or other known liver diseases. MASLD is the most common liver disease in adolescents with obesity. The aims of this study were as follows: (i) to determine which index (waist circumference BMI, WHtR, VAI, METS-IR, METS-VF, HSI, FLI, or MetS_zscore) best explains the prevalence of MASLD in adolescents with obesity; (ii) to determine whether there was a specific index that was most strongly associated with MASLD; (iii) to assess which liver function indexes were most strongly correlated with MASLD. **Methods:** A total of 758 adolescents with severe obesity (BMI z-score > 2) admitted at the Division of Auxology, Istituto Auxologico Italiano, IRCCS, Piancavallo-Verbania for a 3-week multidisciplinary body weight reduction program were selected. Anthropometric parameters (stature, body mass, BMI, and waist and hip circumference) were collected, and body composition (lean and fat mass) was determined using the tetrapolar bioimpedance analysis (BIA) technique. Aspartate aminotransferase (AST), alanine aminotransferase (ALT), gamma-glutamyl transferase (gamma GT), alkaline phosphatase (ALP), bilirubin, glucose, total cholesterol, high-density lipoprotein cholesterol (HDL-C), low-density lipoprotein cholesterol (LDL-C), very low-density lipoprotein cholesterol (VLDL-C), triglycerides (TG), and C-reactive protein (CRP) were measured using standard techniques. MASLD was diagnosed based on abdominal ultrasound results. **Results:** WHtR (65.76%) was the most sensitive compared with other indexes. The HSI (AUC: 0.67 (0.63–0.71, 95% CI), *p*-value < 0.05) showed the best performance in predicting MASLD, with the threshold for having MASLD considered at 48.22. The indexes that showed the worst performance in predicting MASLD were the MetS z-score (AUC: 0.56 (0.52–0.60)) and the VAI (AUC: 0.57 (0.52–0.61)). ALT (OR: 2.92 (2.29–3.77); 95% CI) and AST (OR: 2.52 (2.03–3.20)) were the parameters with a stronger correlation with MASLD. **Conclusions:** The most sensitive index for diagnosing MASLD was the WHtR, based exclusively on anthropometric parameters. HSI was the index that correlated the most with MASLD, while the parameters of liver function (ALT and AST) were the most strongly correlated with the disease and its severity.

## 1. Introduction

Metabolic Dysfunction Associated Steatotic Liver Disease (MASLD) is defined as the accumulation of fat in the liver without excessive alcohol consumption or other known liver disease [1].

MASLD affects 25% of the adult population worldwide, with the highest prevalence rates in South America (31%) and the Middle East (32%) and the lowest prevalence in Africa (14%). The prevalence of MASLD doubled between the 1988–1994 and 2005–2008 survey periods. Further, obesity was present in 51% of individuals with MASLD, diabetes mellitus was found in 23% of MASLD cases, the prevalence of metabolic syndrome in patients with MASLD was 41%, and it was 69% in hyperlipidemia/dyslipidemia [2].

MASLD was diagnosed based on abdominal ultrasound findings. First, the subjects’ ultrasound images were taken by an ultrasound technician. Then, gastroenterology experts evaluated the following ultrasound image features and made the final diagnosis, including hepatorenal echo contrast (0–4 points), depth attenuation (0–2 points), liver brightness (0–4 points), and vascular blurring (0–2 points) [3]. The prevalence of MASLD is higher in males than in females and progressively increases with BMI category [3,4].

Further, MASLD is considered the most common liver disease in adolescents [5]. Although simple steatosis has a benign prognosis, non-alcoholic steatohepatitis (NASH) with advanced histopathology can lead to cirrhosis [5,6].

In adolescents with obesity, the prevalence of MASLD is between 20 and 77% [7], and obesity, hyperglycemia, and insulin resistance are risk factors for MASLD [8,9]. The severity of fatty liver correlated positively with anthropometric measures, including BMI, waist and hip circumference, underbust fold thickness, markers of insulin resistance (QUICKI and Homeostasis Model Assessment (HOMA)), and hypertriglyceridemia [7]. In addition, a multivariate regression analysis showed that BMI and elevated alanine aminotransferase (ALT) were positively associated with fatty liver [7]. Since alcohol consumption and liver infections are rare in adolescents [10], fatty liver in adolescents is mainly due to MASLD. Furthermore, Yoo et al. [11] found an association between MASLD and insulin resistance in Korean children aged 9 to 12 years, especially in boys [11].

Diagnostic or prognostic biomarkers are crucial to limit the occurrence of MASLD. Obesity is known to be one of the most important modifiable risk factors for MASLD, and, especially, the amount of visceral adipose tissue (VAT) is closely related to the occurrence and development of MASLD [10,12]. Therefore, accurately determining VAT is crucial for diagnosing and treating MASLD. Unfortunately, the high cost and technical difficulties of measuring VAT using magnetic resonance imaging make it necessary to find more straightforward and cheaper VAT markers for clinical purposes. Several VAT markers have been identified based on simple anthropometric measurements, such as waist circumference, waist-to-hip ratio (WHR), body mass index (BMI), waist-to-height ratio (WHtR), or even based on specific biochemical parameters, such as visceral adiposity index (VAI), Metabolic Visceral Adiposity Index (METS-VF), steatosis index (HSI) and fatty liver index (FLI), metabolic score of insulin resistance (METS-IR) [13]. Kuang et al. [13] found that METS-VF was a better biomarker for diagnosing MASLD than other surrogate markers for VAT, especially in young women.

The presence of these various indices related to VAT and, thus, the possible presence of MASLD makes it imperative to assess the reliability of the above indexes in identifying adolescents with high-grade obesity affected by MASLD.

The identification of one or more simple instruments capable of detecting the presence of MASLD and metabolic syndrome would make it possible to comprehensively assess the pediatric population with severe obesity, reducing the number of examinations and optimising the resources to be used by identifying the subset of patients who deserve intensive and early treatment to prevent or slow down the development of complications in early life.

The objectives of the present study were, therefore, the following: i. to determine which index (waist circumference, BMI, WHtR, VAI, METS-IR, METS-VF, HSI, FLI, MetS_zscore) best explains the prevalence of MASLD in adolescents with obesity; ii. to determine whether there was a specific index that was the most strongly associated with MASLD; iii. to assess which parameters of liver function were the most strongly correlated with MASLD.

## 2. Materials and Methods

### 2.1. Participants

A retrospective cohort study based on 758 adolescents (age: 14.8 ± 2.1 years; Tanner stage: 3.8 ± 1.4; stature: 1.63 ± 0.10 m; BM: 101.6 ± 22.7 Kg; BMI: 37.9 ± 6.2 Kg m^−2^) with severe obesity (BMI z-score > 2, according to the Italian reference growth charts for age and sex [14]) admitted to the Division of Auxology, Istituto Auxologico Italiano, IRCCS, Piancavallo-Verbania, for a 3-week multidisciplinary body weight reduction program.

Inclusion criteria were as follows: i. age > 10 and <19 years; ii. BMI SDS ≥ 2.0 for sex and age, using the Italian reference curves [15]; iii. essential obesity; iv. abstinence from alcohol; iv. unbalanced type 2 diabetes mellitus. The exclusion criteria were as follows: i. genetic or syndromic obesity; ii. alcohol intake (any quantity); iii. hepatitis B virus or hepatitis C virus infection; iv. type 1 diabetes mellitus.

This study was conducted following the Declaration of Helsinki and approved by the Ethics Committee number 5—Lombardy region (approval number 215/24; research code: 01C411; acronym: NAFLDOB).

At admission to the hospital, informed consent was obtained from all the participants and their parents involved in the study.

### 2.2. Measurements

#### 2.2.1. Physical Characteristics and Body Composition

The subjects’ medical history and physical examination were performed on admission to the hospital. Stature was measured closest to 0.5 cm using a standardised Harpenden stadiometer (Holtain Ltd., Crymych, Dyfed, UK). Body mass (BM) was measured closest to 0.1 kg using an electronic scale (Selus, Italy), with the subjects wearing only light underwear. Body mass index (BMI) was calculated as BM (kg) divided by stature squared (m^2^) [14].

The hip circumference (HC) was measured at the point of maximum posterior protrusion [16]. Waist circumference (WC) was measured in a standing position at the midpoint of the last rib and the iliac crest after a slight exhalation using a non-elastic, flexible tape measure [16].

Body composition was determined using a multifrequency tetrapolar bioelectrical impedance analyser (BIA, Human-IM Scan, DS-Medigroup, Milan, Italy), applying a current of 800 µA at a frequency of 50 kHz. Following the method described by Lukaski [17], the subjects rested in the supine position for 20 min before measurement, with their arms and legs relaxed and avoiding contact with other body parts. To minimise measurement errors, efforts were made to standardise the variables influencing the measurements’ validity, reproducibility, and precision. Fat-free mass (FFM) was estimated using the prediction equation proposed by Bedogni et al. [18]. Fat mass (FM) was determined as the difference between body mass and fat-free mass.

#### 2.2.2. Blood Pressure Measurements

Diastolic and systolic blood pressure (BP) was measured using a standard mercury sphygmomanometer to the nearest 2 mmHg after 5 min of rest. The average of three measurements taken on different days was used. Blood pressure was assessed according to the IDF criteria for pediatric age [19].

#### 2.2.3. Laboratory Analyses

Baseline blood samples were collected via venipuncture following a 12 h overnight fast on the second day of hospitalisation. Aspartate aminotransferase (AST), alanine aminotransferase (ALT), gamma-glutamyl transferase (gamma GT), alkaline phosphatase (ALP), bilirubin, glucose, total cholesterol, high-density lipoprotein cholesterol (HDL-C), low-density lipoprotein cholesterol (LDL-C), very low-density lipoprotein cholesterol (VLDL-C), triglycerides (TG), and C-reactive protein (C-RP) were measured using standard techniques.

#### 2.2.4. Anthropometric and Visceral Adiposity Indexes

MASLD is diagnosed based on abdominal ultrasound findings and HSI and FLI values. Subjects with an HSI > 36 or an FLI ≥ 60 were classified as having MASLD [19].

The following indexes were calculated according to the following formulas:

WHtR [20]: WC (cm)/height (cm);

VAI [21]: (WC (cm)/36.58 + 1.89 × BMI (kg m^−2^)) × (TG (mmol L−1)/0.81 × 1.52/HDL (mmol L^−1^)) female; (WC (cm)/39.68 + 1.88 × BMI (kg m^−2^)) × (TG (mmol L^−1^)/1.03 × 1.31/HDL (mmol L^−1^)) male;

METS-IR [22] = Ln [2 × glycemia (mg/dL)  +  triglycerides (mg/dL)] × BMI/Ln HDL-C (mg/dL);

METS-VF [23] = 4.466 + 0.011 × [(Ln ((Ln (2 × FPG (mmol L^−1^) + TG (mmol L^−1^)) × BMI (kg m^−2^))/(Ln (HDL − C (mmol L^−1^)))))3] + 3.239 × [(Ln(WHtR))3 + 0.319 × sex + 0.594 × [Ln (age)];

HSI [24]: 8 × (ALT (U L^−1^)/AST (U L^−1^)) + BMI (kg m^−2^) (+ 2, if women or diabetes);

FLI [25]: = 100 × EXP(0.953 × Ln(TG (mmol L^−1^)) + 0.139 × BMI (kg m^−2^) + 0.718 × Ln(GGT (U L^−1^)) + 0.053 × WC (cm) − 15.745)/[1 + EXP(0.953 × Ln(TG (mmol L^−1^)) + 0.139 × BMI (kg m^−2^) + 0.718 × Ln(GGT (U L^−1^)) + 0.053 × WC (cm) − 15.745)].

where WC is waist circumference, BMI is body mass index, TG is triglycerides, and FPG is glycemia.

#### 2.2.5. Liver Function Indexes

AST, ALT, gamma GT, bilirubin, ALP, and C-RP were considered as liver function indexes.

### 2.3. Statistical Analyses

The data are expressed as mean and 95% confidence interval (CI). Receiver Operating Characteristic (ROC) curves were created to evaluate the performance of each index as a predictor of hepatic steatosis. For each index, the area under the curve (AUC) and the corresponding 95% CI were calculated along with sensitivity and specificity. Additionally, key diagnostic metrics, including positive predictive value (PPV), negative predictive value (NPV), positive likelihood ratio (PLR), and negative likelihood ratio (NLR), were computed to assess the reliability of each index in identifying hepatic steatosis. The optimal cut-off (threshold) for predicting MASLD was determined by maximising specificity and sensitivity. The ROC analysis also determined the most potent predictor among the adiposity and body composition indexes for metabolic risk factors. The optimal cutoff values for the indexes were identified using the Youden index. The comparison between the AUC of the different indexes was performed using DeLong’s test.

Then, we calculated adjusted Odds Ratios (ORs) using a logistic regression model to assess the relationship between the odds of hepatic steatosis and different indexes. In this analysis, the dependent variable was the presence or absence of hepatic steatosis, while the independent variables were the indexes. Age was included as a covariate to control for its potential confounding effect. The analysis was conducted separately for boys and girls, with age adjustments applied in each group (reported in the Supplementary Material). This approach allowed us to evaluate the strength and direction of the association between each index and the odds of hepatic steatosis while accounting for age-related differences among participants. ORs were deemed statistically significant when the 95% confidence interval (CI) excluded 1. All analyses were conducted using R software, version 4.4 (R Foundation for Statistical Computing). The “pROC” package was used to compute the ROC and the key diagnostic metrics, and the “odds ratio” package was used to assess the OR.

## 3. Results

The descriptive characteristics of the sample are shown in Table 1. Boys and girls had different anthropometric characteristics, with girls weighing 10% less (*p* < 0.001), being 4% smaller (*p* < 0.001), and, thus, having a lower BMI z score (3.33%, *p* = 0.031) than boys. In addition, girls had lower systolic and diastolic blood pressure (3.7%, *p* < 0.001 and 2.3%, *p* < 0.001) than boys. The WHtR, HIS, and FLI indexes were lower in girls (2.9%, 5.6% and 60.8%, *p* < 0.001), while the VAI and the MetS z-score were higher in girls (+15.8 and +16.6%, *p* < 0.01) than boys.

**Figure 1:**
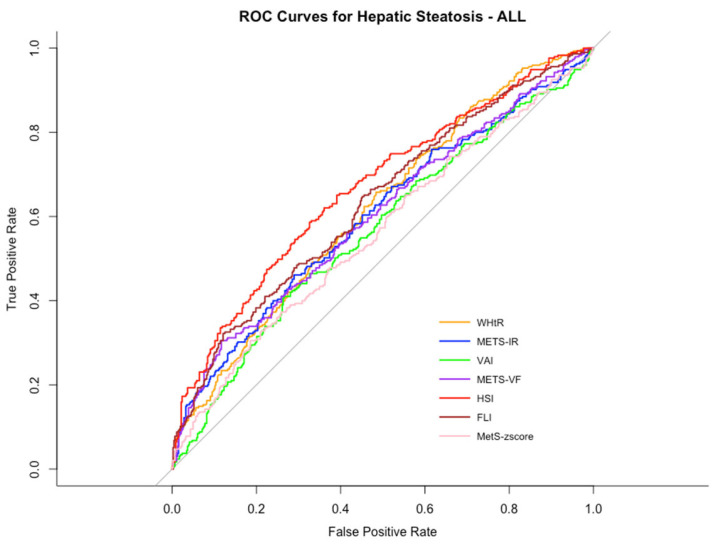
The receiver operating characteristic (ROC) curve of seven anthropometric indexes in predicting hepatic steatosis among male and female adolescents with obesity. Abbreviations: WHtR: waist-to-height ratio; METS-IR: metabolic score of insulin resistance; VAI: visceral adiposity index; METS-VF: metabolic visceral fat score; HSI: hepatic steatosis index; FLI: fatty liver index; MetS_zscore: metabolic syndrome zscore.

Figure 1 shows the ROC, while Table 2 reports the difference between the AUC of the different indexes. The HSI (AUC: 0.67 (0.63–0.71, 95% CI), *p*-value < 0.05) showed the best performance in predicting MASLD, with the threshold for having MASLD considered at 48.22. The indexes that showed the worst performance in predicting MASLD were the MetS z-score (AUC: 0.56 (0.52–0.60)) and the VAI (AUC: 0.57 (0.52–0.61)).

Table 3 shows the performance metrics of the indexes for identifying MASLD. Sensitivity is the ability to detect sick people in a test population. Specificity is the probability of a negative result in definitely healthy subjects. PPV is the probability that a test-positive subject is actually ill. NPV is the probability that people with a negative test result do not actually suffer from the disease or condition in question. The Youden index is used to evaluate the effectiveness of a diagnostic test. PLR and NLR are, respectively, the probability of a positive and negative diagnosis.

WHtR (65.76%) and HIS (58.64%) were the most sensitive compared with other indexes, whereas METS-VF (30.51%) and METS z-score (30.51%) had the lowest sensitivity.

METS-VF (88.34%), FLI (87.90%) and METS z-score (81.43%) showed the highest specificity, and WHtR (51.62%) was the lowest.

METS-VF (62.50%) and FLI (62.91%) showed higher PPV, HIS (71.89%) and WHtR (70.29%) showed higher NPV compared with other indexes.

The Youden index was higher for HIS (0.26), PLR was higher in FLI (2.66) and METS-VF (2.62) compared with the other indexes, and NLR was higher for the METS z-score (0.85) than for the other indexes.

**Figure 2:**
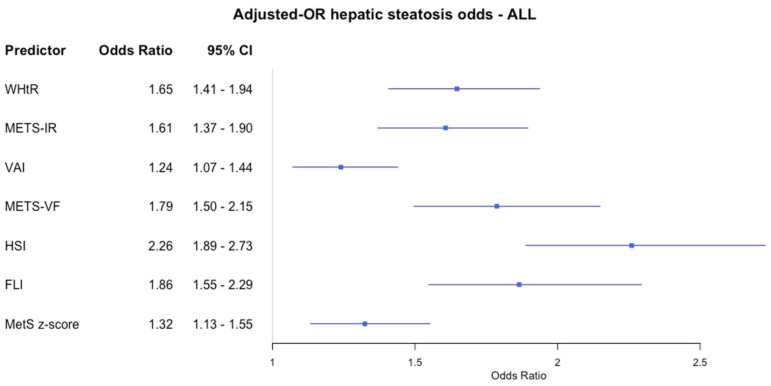
Forest plot of adjusted odds ratios (ORs) and 95% confidence intervals (CIs) for indexes of hepatic steatosis in adolescents with obesity. Each row displays a specific index, with the square dot representing the adjusted-for-age odds ratio and the horizontal line extending from the dot indicating the 95% confidence interval. The plot includes a vertical reference line at an OR of 1.0, representing no effect. Predictors with confidence intervals that do not cross this line suggest a statistically significant association with obesity risk. The numerical values of the odds ratio values and their CI are placed next to each index. Abbreviations: WHtR: waist-to-height ratio; METS-IR: metabolic score of insulin resistance; VAI: visceral adiposity index; METS-VF: metabolic visceral fat score; HSI: hepatic steatosis index; FLI: fatty liver index; MetS_zscore: metabolic syndrome zscore.

Figure 2 shows how the different visceral adiposity indexes affect the odds of having hepatic steatosis in adolescents with obesity, adjusting all for age. All the indexes were directly related to the odds of having hepatic steatosis. However, the strength of this association was stronger for the HSI (OR: 2.26 (1.89–2.73); 95% CI) than the other indexes, with a 1 SD increment in HSI corresponding to a 126% increase in the odds. VAI (OR: 1.27 (1.07–1.44)) and BMI z-score (OR: 1.32 (1.13–1.55)) had a weaker link to the odds of having hepatic steatosis.

**Figure 3:**
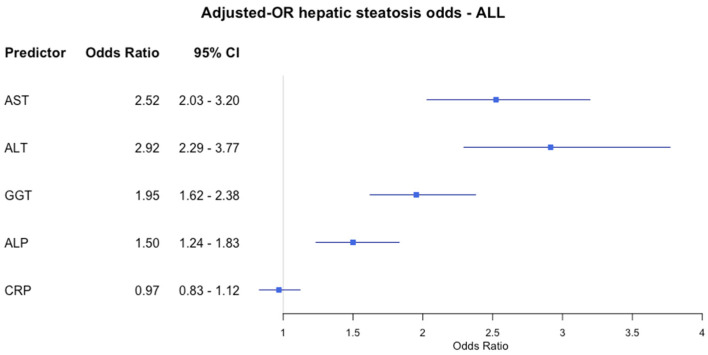
Forest plot of adjusted odds ratios (ORs) and 95% confidence intervals (CIs) for predictors of hepatic steatosis in adolescents with obesity. Each row displays a specific index, with the square dot representing the adjusted-for-age odds ratio and the horizontal line extending from the dot indicating the 95% confidence interval. The plot includes a vertical reference line at an OR of 1.0, representing no effect. Predictors with confidence intervals that do not cross this line suggest a statistically significant association with obesity risk. The numerical values of the odds ratio values and their CI are placed next to each index. Abbreviation: AST: Aspartate aminotransferase; ALT: alanine aminotransferase; GGT: gamma-glutamyl transferase; ALP: alkaline phosphatase; CRP: C-reactive protein.

Figure 3 shows how the liver function indexes affect the odds of having hepatic steatosis in adolescents with obesity, adjusting all for age. All the indexes, except for the C-RP, were directly related to the odds of having hepatic steatosis. However, the strength of this association was stronger for the ALT (OR: 2.92 (2.29–3.77); 95% CI) and the AST (OR: 2.52 (2.03–3.20)).

## 4. Discussion

This study investigated which index correlates better with the diagnosis of hepatic steatosis in a group of 758 adolescents of both sexes. Of all the indexes considered, HSI was the best, followed by FLI, WHtR, Mets-IR and Mets-VF, Mets z-score, and VAI. WHtR was the most sensitive index for diagnosis of MASLD, and ALT and AST indexes were the most strongly correlated with MASLD.

In the present study, steatosis was found in 162 boys and 133 girls, i.e., in 21.4 and 17.5% of those examined. If we consider only the boys, 53.3% of those measured had the disease, and if we consider only the girls, 29.3% had the disease. This confirms the finding of Zhang et al. [26] that the prevalence of MASLD has increased by 1.35 per year since 1990, with the increase being greater in boys than in girls. In addition, previous studies [24,25,27,28] showed that the main risk factors for MASLD in adolescents were insulin resistance and central obesity, which is consistent with the results of the present study.

The HSI index was the best predictor of MASLD, as shown by Sheng et al. [25] and confirmed in the present study. Also, according to [29], HSI showed a better predictive performance for MASLD than BMI, WHtR, lipid accumulation product (LAP), body roundness index, conicity index (COI), VAI, TyG index, waist-to-hip ratio, body adiposity index (BAI), and abdominal volume index (AVI) [29]. However, in elderly people, Zhang et al. [26] showed that LAP was the best marker for predicting MASLD compared to relative fat mass, WC, Body Shape Index, WHtR, COI, Ponderal Index, and BMI.

FLI index is not only associated with MASLD but also with hypertension-induced organ damage (HMOD) in never-treated hypertensives without diabetes mellitus. Adolescents at high risk of MASLD were 2.3 times more likely to have HMOD compared to their low-risk peers, and the FLI index was also linearly correlated with the presence of HMOD [30].

In the present study, we showed that WHtR was the most sensitive index for the diagnosis of MASLD, an index based solely on anthropometric parameters, which anyone can determine without blood tests or ultrasound examinations. According to Sheng et al. [31], WHtR was one of the most sensitive indexes (i.e., sensitivity: 0.83) for detecting MASLD. Furthermore, high WHtR values were significantly associated with the risk of MASLD [24].

In addition, our data showed that higher ALT and AST levels were strongly correlated with MASLD. This is consistent with the findings of Mischel et al. [32], where higher ALT levels were observed in adolescents with obesity and a higher prevalence of MASLD. Xuan et al. [33] also showed in a study with 4753 participants that a new simple and non-invasive biomarker was the ALT/AST ratio. This was consistent with our results, which showed that both ALT and AST were strongly correlated with disease. When the standard deviation of these parameters increased, the percentage risk of developing the disease increased [33].

Most children with MASLD showed clinical symptoms between the ages of 10 and 13 years [34] with different histopathologic features compared to adults [35]. Dong et al. [36] also found that fatty degeneration was more pronounced in children than in adults, and AST and ALT levels reflected the histopathologic severity of MASLD. Thus, ALT and AST levels were not only highly correlated with MASLD but were also predictors of disease severity in adolescents [36], as shown by the odds ratios of our study. Therefore, physicians could use simpler and less invasive indexes such as HSI, ALT, and AST, as well as the ALT/AST ratio in adolescence, to improve prognosis and prevent disease progression through lifestyle changes or medication.

In summary, the most sensitive index for the diagnosis of MASLD was the WHtR, an index based solely on anthropometric parameters. HSI was the index of obesity most strongly correlated with MASLD, and liver function parameters (ALT and AST) were most strongly correlated with the disease and its severity.

## Figures and Tables

**Table 1:** Descriptive characteristics and comparisons between girls and boys with obesity.

	Girls (n = 454)	Boys (n = 304)	*p*-Value
Age	14.8 (14.6, 15.0)	14.6 (14.4, 14.9)	0.209
Body weight (kg)	97.2 (95.5, 98.9)	108.1 (105.2, 111.1)	<0.001
Height (m)	1.6 (1.6, 1.6)	1.7 (1.7, 1.7)	<0.001
Body mass index (kg m^−2^)	37.7 (37.1, 38.2)	38.3 (37.6, 39.0)	0.188
Body mass index z-score	3.0 (2.9, 3.0)	3.1 (3.0, 3.2)	0.031
Waist Circumference (cm)	112.0 (110.8, 113.2)	120.1 (118.4, 121.8)	<0.001
Hip Circumference (cm)	122.3 (121.2, 123.3)	120.7 (119.2, 122.2)	0.055
Systolic (mmHg)	123.5 (122.4, 124.6)	128.4 (126.9, 129.8)	<0.001
Diastolic (mmHg)	77.8 (77.1, 78.5)	79.7 (78.7, 80.6)	<0.001
AST (U L^−1^)	20.2 (19.4, 21.0)	26.5 (25.3, 27.7)	<0.001
ALT (U L^−1^)	23.8 (22.3, 25.4)	38.5 (35.2, 41.7)	<0.001
GGT (U L^−1^)	16.3 (15.5, 17.0)	24.1 (22.6, 25.7)	<0.001
ALP (U L^−1^)	112.7 (107.4, 118.0)	190.4 (180.3, 200.6)	<0.001
Bilirubin (μmol L^−1^)	0.6 (0.6, 0.6)	0.7 (0.7, 0.7)	0.002
Fasting glucose (mg dL^−1^)	81.2 (80.6, 81.8)	81.9 (81.3, 82.6)	0.047
Total cholesterol (mg dL^−1^)	162.8 (159.9, 165.6)	165.3 (161.7, 169.0)	0.318
HDL-C (mg dL^−1^)	44.3 (43.3, 45.3)	40.5 (39.3, 41.7)	<0.001
LDL-C (mg dL^−1^)	101.729 (99.1, 104.4)	107.1 (103.9, 110.3)	0.009
VLDL-C (mg dL^−1^)	18.6 (17.8, 19.3)	20.6 (19.6, 21.6)	<0.001
Tryglicerides (mg dL^−1^)	93.1 (89.4, 96.8)	101.8 (97.1, 106.5)	<0.001
C-reactive protein (mg dL^−1^)	0.57 (0.51, 0.63)	0.57 (0.47, 0.66)	0.481
WHtR	0.70 (0.69, 0.71)	0.72 (0.71, 0.73)	<0.001
VAI (cm^−2^)	1.9 (1.8, 2.0)	1.6 (1.5, 1.8)	0.003
METS-IR	783.3 (771.2, 795.4)	781.6 (766.4, 796.9)	0.758
METS-VF	9.4 (9.4, 9.5)	9.5 (9.4, 9.5)	0.257
HSI	46.6 (46.0, 47.3)	49.2 (48.2, 50.1)	<0.001
FLI	7.4 (6.1, 8.7)	11.9 (9.8, 13.9)	<0.001
MetS_zscore	1.6 (1.5, 1.6)	1.3 (1.2, 1.4)	<0.001

Data are presented as mean (95% confidence interval). Two-sided *p* values were obtained from an analysis of covariance comparing girls vs. boys adjusting for age. Abbreviations: AST: Aspartate aminotransferase; ALT: alanine aminotransferase; GT: gamma-glutamyl transferase; ALP: alkaline phosphatase; HDL-C: high-density lipoprotein cholesterol; LDL-C: low-density lipoprotein cholesterol; VLDL-C: very low-density lipoprotein cholesterol; WHtR: waist-to-height ratio; VAI: visceral adiposity index; METS-IR: metabolic score of insulin resistance; METS-VF: metabolic visceral fat score; HSI: hepatic steatosis index; FLI: fatty liver index; MetS_zscore: metabolic syndrome zscore.

**Table 2:** The area under the curve (AUC) of the receiving operator curve (ROC) for adiposity indexes and hepatic steatosis in adolescents with obesity.

	AUC (95% CI)	*p* vs. WHtR	*p* vs. METS-IR	*p* vs. VAI	*p* vs. METS-VF	*p* vs. HSI	*p* vs. FLI
WHtR	0.62 (0.58–0.66)						
METS-IR	0.60 (0.56–0.64)	0.344					
VAI	0.57 (0.52–0.61)	0.061	0.259				
METS-VF	0.60 (0.56–0.64)	0.264	0.843	0.212			
HSI	0.67 (0.63–0.71)	0.003	<0.001	<0.001	<0.001		
FLI	0.63 (0.59–0.67)	0.248	0.011	0.006	<0.001	0.002	
MetS_zscore	0.56 (0.52–0.60)	0.014	0.080	0.795	0.054	<0.001	<0.001

Abbreviations: WHtR: waist-to-height ratio; METS-IR: metabolic score of insulin resistance; VAI: visceral adiposity index; METS-VF: metabolic visceral fat score; HSI: hepatic steatosis index; FLI: fatty liver index; MetS_zscore: metabolic syndrome zscore.

**Table 3:** Diagnostic performance metrics of anthropometric indexes for identifying hepatic steatosis in adolescents with obesity.

	Threshold	Sensitivity (%)	Specificity (%)	PPV (%)	NPV (%)	Youden Index	PLR	NLR
WHtR	0.69	65.76	51.62	46.41	70.29	0.17	1.36	0.66
METS-IR	807.93	46.10	71.06	50.37	67.42	0.17	1.59	0.76
VAI	1.92	41.02	73.43	49.59	66.15	0.14	1.54	0.80
METS-VF	9.75	30.51	88.34	62.50	66.61	0.19	2.62	0.79
HSI	48.22	58.64	67.39	53.40	71.89	0.26	1.80	0.61
FLI	11.78	32.20	87.90	62.91	67.05	0.20	2.66	0.77
MetS_zscore	1.86	30.51	81.43	51.14	64.78	0.12	1.64	0.85

Abbreviation: WHtR: waist-to-height ratio; METS-IR: metabolic score of insulin resistance; VAI: visceral adiposity index; METS-VF: metabolic visceral fat score; HSI: hepatic steatosis index; FLI: fatty liver index; MetS_zscore: metabolic syndrome zscore.

## Data Availability

Raw data will be uploaded to www.zenodo.org immediately after the acceptance of the manuscript (accessed on 5 January 2025) and will be available upon reasonable request to the authors S.L. and A.S.

## Supplementary Materials

The following supporting information can be downloaded at https://www.mdpi.com/article/10.3390/jcm14062085/s1, Table S1: The area under the curve (AUC) of the receiving operator curve (ROC) for adiposity indexes and hepatic steatosis in boys with obesity, Table S2: Diagnostic performance metrics of anthropometric indexes for identifying hepatic steatosis in boys with obesity, Table S3: The area under the curve (AUC) of the receiving operator curve (ROC) for adiposity indexes and hepatic steatosis in girls with obesity, Table S4: Diagnostic performance metrics of anthropometric indexes for identifying hepatic steatosis in girls with obesity, Figure S1: The receiver operating characteristic (ROC) curve of seven anthropometric indexes in predicting hepatic steatosis among boys with obesity, Figure S2: The receiver operating characteristic (ROC) curve of seven anthropometric indexes in predicting hepatic steatosis among girls with obesity, Figure S3: Forest plot of adjusted odds ratios (ORs) and 95% confidence intervals (CIs) for predictors of hepatic steatosis in boys with obesity, Figure S4: Forest plot of adjusted odds ratios (ORs) and 95% confidence intervals (CIs) for predictors of hepatic steatosis in girls with obesity, Figure S5: Forest plot of adjusted odds ratios (ORs) and 95% confidence intervals (CIs) for predictors of hepatic steatosis in boys with obesity, Figure S6: Forest plot of adjusted odds ratios (ORs) and 95% confidence intervals (CIs) for predictors of hepatic steatosis in girls with obesity.

## Abbreviations

The following abbreviations are used in this manuscript:

MASLD	Metabolic dysfunction associated steatotic liver disease
NASH	Non-alcoholic steatohepatitis
BMI	Body mass index
HOMA	Homeostasis Model Assessment
ALT	Alanine aminotransferase
VAT	Visceral adipose tissue
WHR	Waist-to-hip ratio
WHtR	Waist-to-height ratio
VAI	Visceral Adiposity Index
METS-VF	Metabolic Visceral Adiposity Index
METS-IR	Metabolic Score of Insulin Resistance
HIS	Steatosis Index
FLI	Fatty Liver Index
BP	blood pressure
AST	Aspartate aminotransferase
ALT	Alanine aminotransferase
ALP	Alkaline phosphatase
HDL-C	High-density lipoprotein cholesterol
LDL-C	Low-density lipoprotein cholesterol
VLDL-C	Very low-density lipoprotein cholesterol
TG	Triglycerides
C-RP	C-reactive protein
BM	Body mass
HC	Hip circumference
WC	Waist circumference
BIA	Bioelectrical impedance analyser
FM	Fat mass
FFM	Fat free mass
