# Supplementary material for: Visceral Adiposity, Anthropometric and Liver Function Indexes for Identifying Metabolic Dysfunction Associated Steatotic Liver Disease (MASLD) in Adolescents with Obesity: Which Performs Better?
Source: J Clin Med. 2025 Mar 19;14(6):2085. doi: 10.3390/jcm14062085 (PMC11943388; doi:10.3390/jcm14062085)
Supplement: Supplementary file 1 [file jcm-14-02085-s001.zip › jcm-3491474-supplementary.pdf]

Journal of Clinical Medicine

Supplementary Materials

Table S1. The area under the curve (AUC) of the receiving operator curve (ROC) for adiposity indexes and hepatic steatosis in boys with obesity.

	AUC	p vs WHtR	p vs METS_IR	p vs VAI	p vs METS_VF	p vs HSI	p vs FLI
WHtR	0.57 (0.51-0.64)						
METS-IR	0.61 (0.55-0.68)	0.092					
VAI	0.54 (0.47-0.6)	0.396	0.086				
METS-VF	0.59 (0.53-0.66)	0.297	0.059	0.184			
HSI	0.66 (0.60-0.72)	**0.002**	0.021	**0.003**	**< 0.001**		
FLI	0.59 (0.52-0.65)	0.528	0.118	0.163	0.557	**< 0.001**	
MetS z-score	0.55 (0.49-0.62)	0.602	0.081	0.439	0.214	**< 0.001**	0.183

Abbreviations: WHtR: waist-to-height ratio; METS-IR: metabolic score of insulin resistance; VAI: visceral adiposity index; METS-VF: metabolic visceral fat score; HSI: hepatic steatosis index; FLI: fatty liver index; MetS_zscore: metabolic syndrome zscore.

https://doi.org/10.3390/jcm14062085

Table S2. Diagnostic performance metrics of anthropometric indexes for identifying hepatic steatosis in boys with obesity.

	Threshold	Sensitivity (%)	Specificity (%)	PPV (%)	NPV (%)	Youden_ Index	PLR	NLR
WHtR	0.68	74.69	39.44	58.45	57.73	0.14	1.23	0.64
METS-IR	751.78	58.64	62.68	64.19	57.05	0.21	1.57	0.66
VAI	1.92	32.72	78.17	63.10	50.45	0.11	1.50	0.86
METS-VF	9.59	42.59	75.35	66.35	53.50	0.18	1.73	0.76
HSI	47.34	65.43	61.27	65.84	60.84	0.27	1.69	0.56
FLI	8.19	41.36	73.94	64.42	52.50	0.15	1.59	0.79
MetS z-score	1.82	25.93	85.92	67.74	50.41	0.12	1.84	0.86

Abbreviations: WHtR: waist-to-height ratio; METS-IR: metabolic score of insulin resistance; VAI: visceral adiposity index; METS-VF: metabolic visceral fat score; HSI: hepatic steatosis index; FLI: fatty liver index; MetS_zscore: metabolic syndrome zscore.

Table S3. The area under the curve (AUC) of the receiving operator curve (ROC) for adiposity indexes and hepatic steatosis in girls with obesity.

Predictor	AUC	p vs WHtR	p vs METS_IR	p vs VAI	p vs METS_VF	p vs HSI	p vs FLI
WHtR	0.63 (0.58-0.69)						
METS-IR	0.60 (0.55-0.66)	0.291					
VAI	0.63 (0.57-0.69)	0.980	0.543				
METS-VF	0.61 (0.55-0.67)	0.245	0.662	0.603			
HSI	0.66 (0.60-0.71)	0.257	**0.004**	0.467	**0.009**		
FLI	0.64 (0.58-0.70)	0.676	0.062	0.806	**0.028**	0.226	
MetS z-score	0.63 (0.57-0.69)	0.949	0.402	0.973	0.478	0.222	0.671

Abbreviations: WHtR: waist-to-height ratio; METS-IR: metabolic score of insulin resistance; VAI: visceral adiposity index; METS-VF: metabolic visceral fat score; HSI: hepatic steatosis index; FLI: fatty liver index; MetS_zscore: metabolic syndrome zscore.

Table S4. Diagnostic performance metrics of anthropometric indexes for identifying hepatic steatosis in girls with obesity.

Predictor	Threshold	Sensitivity (%)	Specificity (%)	PPV (%)	NPV (%)	Youden _Index	PLR	NLR
WHtR	0.74	44.36	76.01	43.38	76.73	0.20	1.85	0.73
METS-IR	894.57	29.32	88.47	51.32	75.13	0.18	2.54	0.80
VAI	1.79	57.14	66.04	41.08	78.81	0.23	1.68	0.65
METS-VF	9.75	31.58	88.79	53.85	75.80	0.20	2.82	0.77
HSI	45.82	66.17	58.26	39.64	80.60	0.24	1.59	0.58
FLI	2.64	62.41	60.44	39.52	79.51	0.23	1.58	0.62
MetS z-score	1.70	51.88	68.85	40.83	77.54	0.21	1.67	0.70

Abbreviations: WHtR: waist-to-height ratio; METS-IR: metabolic score of insulin resistance; VAI: visceral adiposity index; METS-VF: metabolic visceral fat score; HSI: hepatic steatosis index; FLI: fatty liver index; MetS_zscore: metabolic syndrome zscore.

Figure S1. The receiver operating characteristic (ROC) curve of seven anthropometric indexes in predicting hepatic steatosis among boys with obesity.

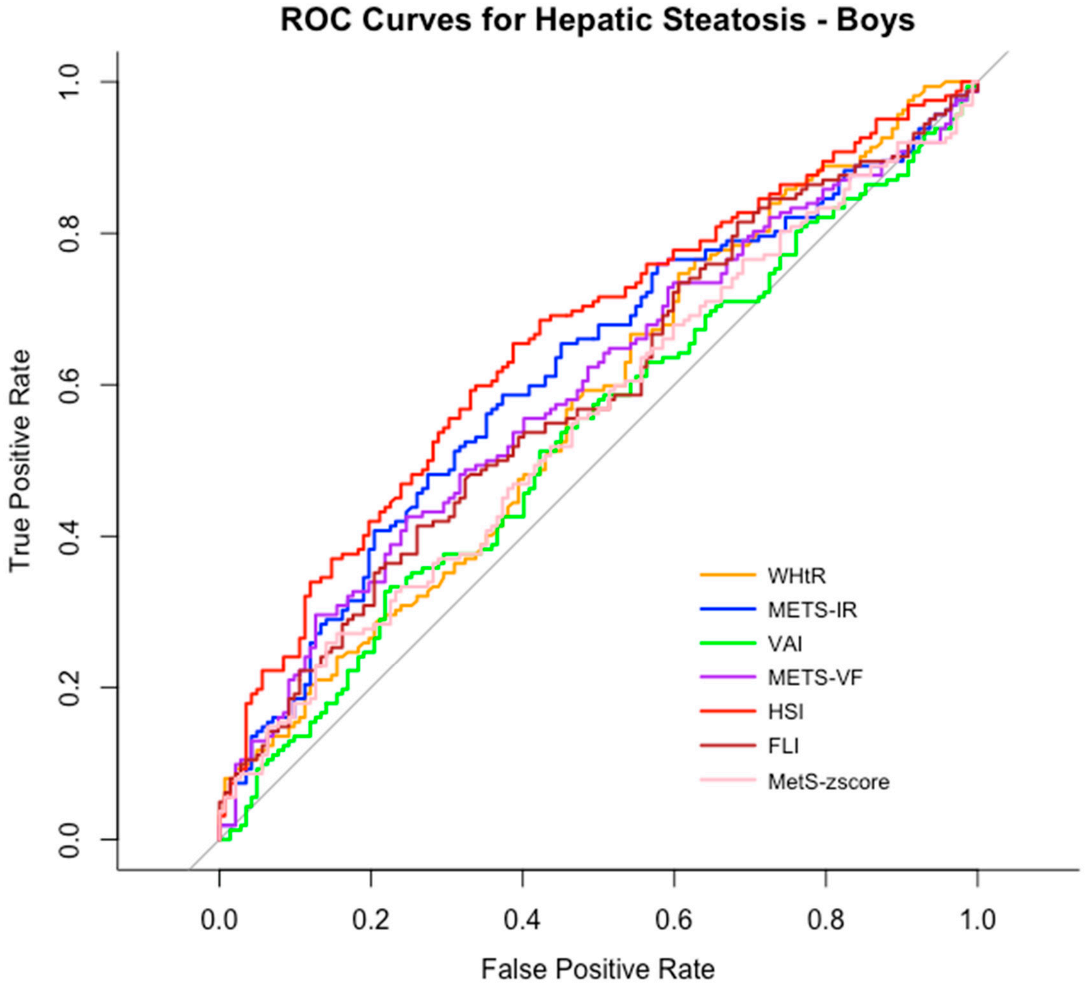

Abbreviations: WHtR: waist-to-height ratio; METS-IR: metabolic score of insulin resistance; VAI: visceral adiposity index; METS-VF: metabolic visceral fat score; HSI: hepatic steatosis index; FLI: fatty liver index; MetS_zscore: metabolic syndrome zscore.

Figure S2. The receiver operating characteristic (ROC) curve of seven anthropometric indexes in predicting hepatic steatosis among girls with obesity.

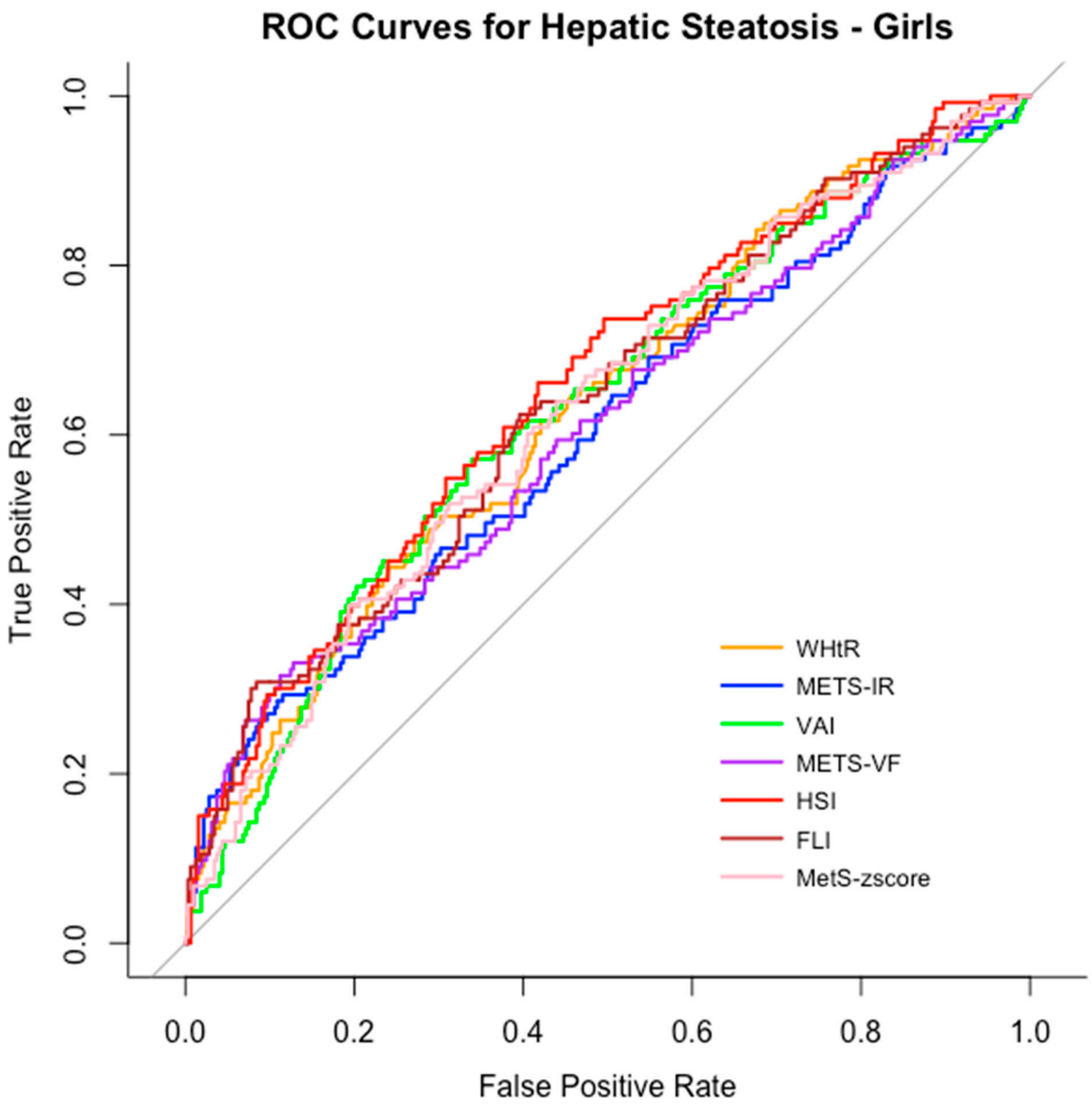

Abbreviations: WHtR: waist-to-height ratio; METS-IR: metabolic score of insulin resistance; VAI: visceral adiposity index; METS-VF: metabolic visceral fat score; HSI: hepatic steatosis index; FLI: fatty liver index; MetS_zscore: metabolic syndrome zscore.

Figure S3. Forest plot of adjusted odds ratios (ORs) and 95% confidence intervals (CIs) for predictors of hepatic steatosis in boys with obesity. Each row displays a specific index, with the square dot representing the adjusted-for-age odds ratio and the horizontal line extending from the dot indicating the 95% confidence interval. The plot includes a vertical reference line at an OR of 1.0, representing no effect. Predictors with confidence intervals that do not cross this line suggest a statistically significant association with obesity risk. The numerical values of the odds ratio values and their CI are placed next to each index.

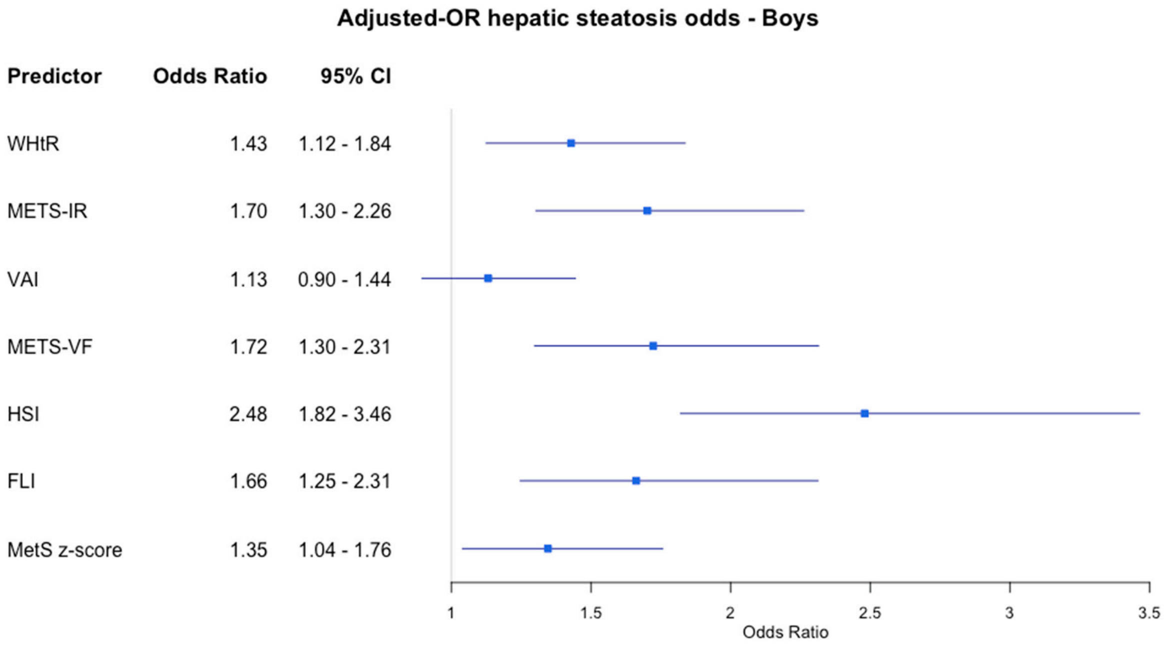

Abbreviations: WHtR: waist-to-height ratio; METS-IR: metabolic score of insulin resistance; VAI: visceral adiposity index; METS-VF: metabolic visceral fat score; HSI: hepatic steatosis index; FLI: fatty liver index; MetS_zscore: metabolic syndrome zscore.

Figure S4. Forest plot of adjusted odds ratios (ORs) and 95% confidence intervals (CIs) for predictors of hepatic steatosis in girls with obesity. Each row displays a specific index, with the square dot representing the adjusted-for-age odds ratio and the horizontal line extending from the dot indicating the 95% confidence interval. The plot includes a vertical reference line at an OR of 1.0, representing no effect. Predictors with confidence intervals that do not cross this line suggest a statistically significant association with obesity risk. The numerical values of the odds ratio values and their CI are placed next to each index.

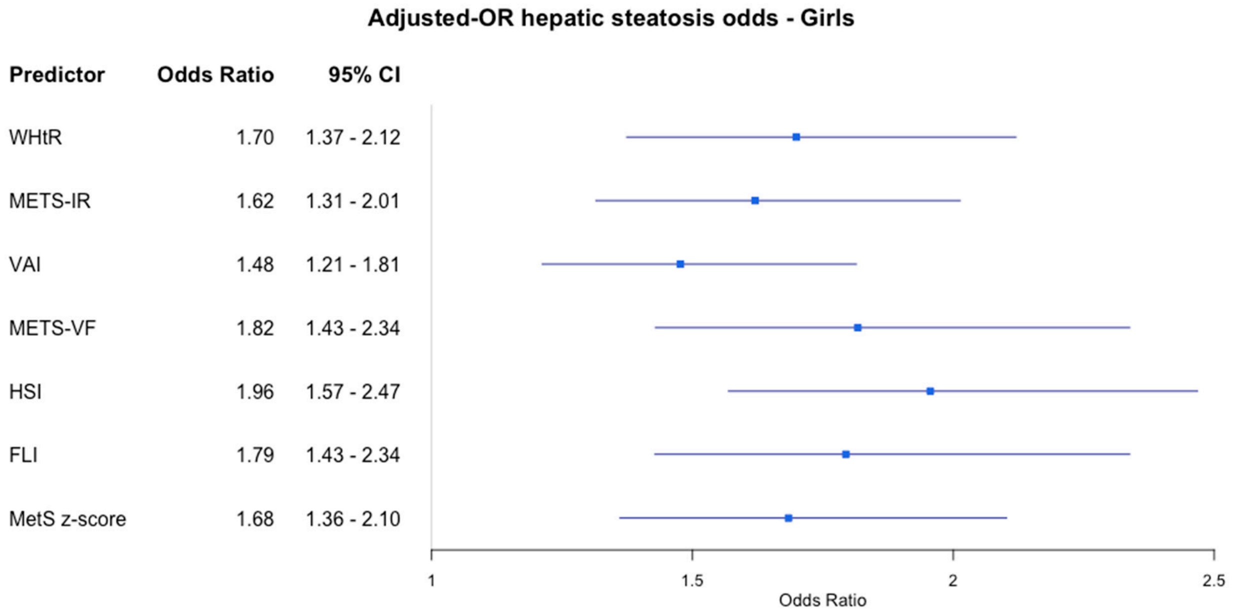

Abbreviations: WHtR: waist-to-height ratio; METS-IR: metabolic score of insulin resistance; VAI: visceral adiposity index; METS-VF: metabolic visceral fat score; HSI: hepatic steatosis index; FLI: fatty liver index; MetS_zscore: metabolic syndrome zscore.

Figure S5. Forest plot of adjusted odds ratios (ORs) and 95% confidence intervals (CIs) for predictors of hepatic steatosis in boys with obesity. Each row displays a specific index, with the square dot representing the adjusted-for-age odds ratio and the horizontal line extending from the dot indicating the 95% confidence interval. The plot includes a vertical reference line at an OR of 1.0, representing no effect. Predictors with confidence intervals that do not cross this line suggest a statistically significant association with obesity risk. The numerical values of the odds ratio values and their CI are placed next to each index.

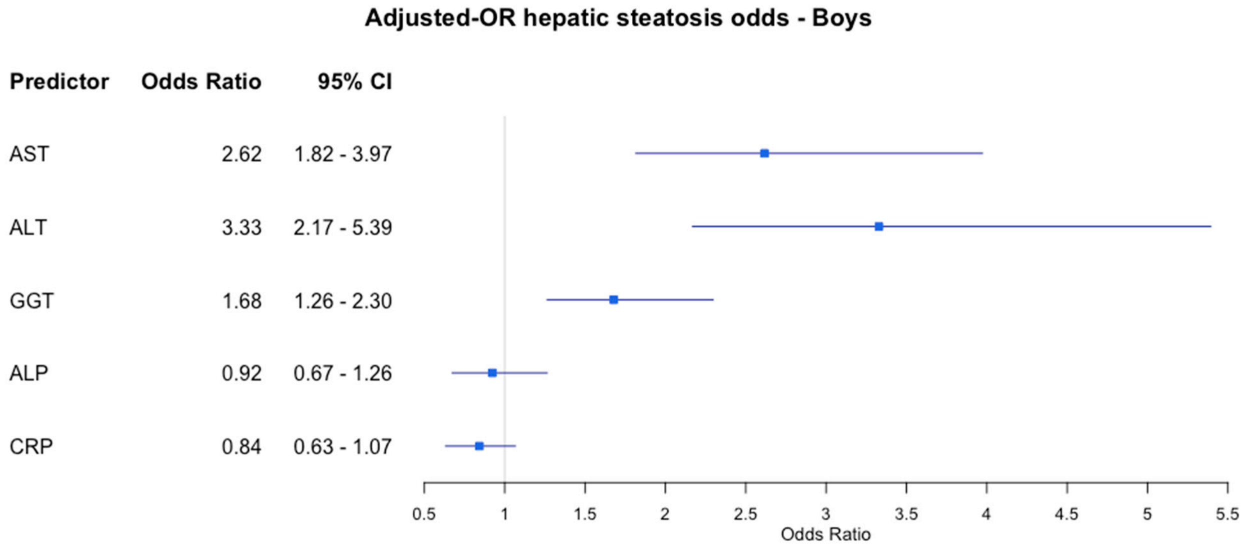

AST: Aspartate aminotransferase; ALT: alanine aminotransferase; GGT: gamma-glutamyl transferase; ALP: alkaline phosphatase; CRP: C-reactive protein

Figure S6. Forest plot of adjusted odds ratios (ORs) and 95% confidence intervals (CIs) for predictors of hepatic steatosis in girls with obesity. Each row displays a specific index, with the square dot representing the adjusted-for-age odds ratio and the horizontal line extending from the dot indicating the 95% confidence interval. The plot includes a vertical reference line at an OR of 1.0, representing no effect. Predictors with confidence intervals that do not cross this line suggest a statistically significant association with obesity risk. The numerical values of the odds ratio values and their CI are placed next to each index.

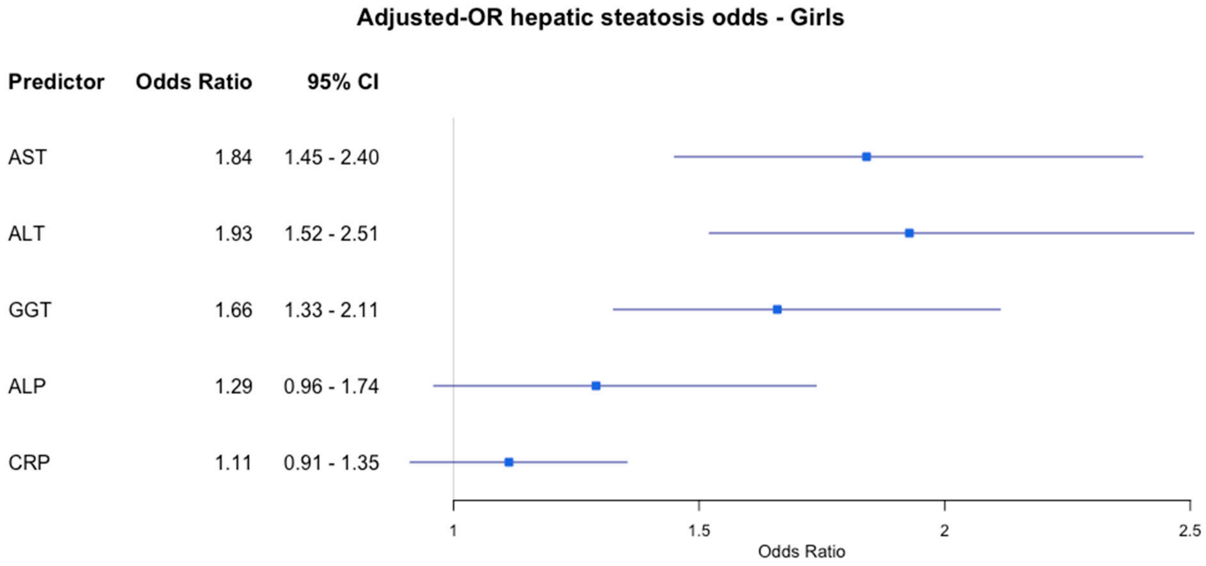

AST: Aspartate aminotransferase; ALT: alanine aminotransferase; GGT: gamma-glutamyl transferase; ALP: alkaline phosphatase; CRP: C-reactive protein
